# Prevalence of paediatric chronic suppurative otitis media and hearing impairment in rural Malawi: A cross-sectional survey

**DOI:** 10.1371/journal.pone.0188950

**Published:** 2017-12-21

**Authors:** Luke Hunt, Wakisa Mulwafu, Victoria Knott, Chifundo B. Ndamala, Andrew W. Naunje, Sam Dewhurst, Andrew Hall, Kevin Mortimer

**Affiliations:** 1 Liverpool School of Tropical Medicine, Liverpool, United Kingdom; 2 College of Medicine, Blantyre, Malawi; 3 Sheffield Teaching Hospitals NHS Foundation Trust, Sheffield, United Kingdom; 4 Malawi-Liverpool-Wellcome Trust Clinical Research Program, Blantyre, Malawi; 5 University Hospitals Leicester NHS Foundation Trust, Leicester, United Kingdom; 6 Independent Scholar, Sheffield, United Kingdom; Telethon Kids Institute, The University of Western Australia, AUSTRALIA

## Abstract

**Objective:**

To estimate the prevalence of World Health Organization-defined chronic suppurative otitis media (CSOM) and mild hearing impairment in a population representative sample of school-entry age children in rural Malawi. A secondary objective was to explore factors associated with CSOM in this population.

**Methods:**

We performed a community-based cross-sectional study of children aged 4–6 years in Chikhwawa District, Southern Malawi, utilising a village-level cluster design. Participants underwent a structured clinical assessment, including video-otoscopy and screening audiometry. Diagnoses were made remotely by two otolaryngologists who independently reviewed clinical data and images collected in the field. Hearing impairment was classified as failure to hear a pure tone of 25dB or greater at 1, 2 or 4kHz.

**Results:**

We recruited 281 children across 10 clusters. The prevalence estimates of CSOM, unilateral hearing impairment and bilateral hearing impairment were 5.4% (95%CI 2.2–8.6), 24.5% (95%CI 16.3–30.0), and 12.5% (95%CI 6.2–16.9) respectively. Middle ear disease was seen in 46.9% of children with hearing impairment. A trend towards increased risk of CSOM was observed with sleeping in a house with >2 other children.

**Interpretation:**

We found a high burden of middle ear disease and preventable hearing impairment in our sample of school-entry age children in rural Malawi. There are important public health implications of these findings as CSOM and hearing impairment can affect educational outcomes, and may impact subsequent development. The identification and management of middle ear disease and hearing impairment represent major unmet needs in this population.

## Introduction

Chronic suppurative otitis media (CSOM) is an infection of the middle ear cleft characterised by perforation of the tympanic membrane and persistent ottorhoea. CSOM is a substantial global health problem with an estimated incidence of about 31 million cases per year.[1,2] Approximately 22.6% of cases occur in children under five.[1]

In sub-Saharan Africa, CSOM is a leading cause of preventable childhood hearing loss.[2,3]. CSOM is independently associated with decreased academic performance and may lead to long-term effects on language and cognitive development.[2,4] Additionally, a substantial proportion of those with untreated CSOM (1–18%) develop serious complications including mastoid abscess, otitic meningitis, venous sinus thrombosis and cholesteatoma.[5,6] Approximately 28000 deaths are attributed to the complications of CSOM annually.[1,2] The complications of CSOM require specialist intervention, frequently unavailable in low-resource healthcare systems.

The recent development of tertiary otolaryngology and audiology services in Southern Malawi along with establishment of a national diploma in otolaryngology for healthcare workers has markedly increased the resources available to manage CSOM and hearing impairment in this setting.[7] Additionally, the development of low-cost smartphone audiometry and video-otoscopy hold promise for linking rural communities with tertiary services.[8–10] However, the prevalence of CSOM and hearing impairment in Malawi are undetermined.

Surveys of CSOM in the sub-Saharan African region have reported broad variation in prevalence estimates, ranging from 1–10%.[4,11–20]. Although this may be explained by varying exposure to disease risk factors between settings, differences in classification of CSOM often limit useful comparison between studies.

The primary objective of this study was to estimate the prevalence of World Health Organization (WHO)-defined CSOM and hearing impairment in a representative sample of rural children in Southern Malawi. A secondary objective was to evaluate potential associations of CSOM in this population. The study was undertaken in communities that had participated in the Cooking and Pneumonia Study (CAPS), a cluster randomized controlled trial of two cleaner-burning biomass-fueled cookstoves (Philips HD4012LS) compared to continuation of open fire cooking on the incidence of childhood pneumonia.[21] Since emissions from these cookstoves are lower than open fires per cooking task and since environmental exposure to household air pollution has been associated with otitis media in a sub-Saharan African settings,[12,22–24] we hypothesised that the prevalence of CSOM would be lower in CAPS intervention group households.

## Materials and methods

### Study design

We conducted a community-based cross-sectional study of children between 4 and 6 years old living in Chikhwawa district in Southern Malawi, and utilised a village level cluster design. The study was based at the Malawi-Liverpool-Wellcome Trust (MLW) clinical research program Chikhwawa field site.

### Study setting

Chikhwawa district (Lat: 16°04′S; Long: 34°80′E) covers an area of 4755Km^2^ with a population of 245,000, of which 45.9% are under 15 years old.[25] The district is exclusively rural with subsistence and smallholder farming the main economic activities.[26] In 2010, 65% of the population lived on less than USD $1.25 per day and the under-five mortality rate was 140/1000.[26] Infrastructure is poor, with regular food shortages due to drought or flood related crop failure.[27]

### Sampling strategy

The sampling frame listed all villages that utilised Chikhwawa District Hospital for primary healthcare needs. This was created by MLW field workers with the District Medical Officer as part of CAPS. We utilised a two-stage sampling strategy. At the first stage, 10 villages were selected from the sampling frame by simple random sampling without replacement. The number of children aged 4–6 years in each of these villages was established through door to door census conducted for the current study. Cluster sizes were determined by dividing the required sample size by the total number of eligible children in each village. At stage two, we subsampled observational units from each cluster using simple random sampling without replacement. All children identified in the initial survey were assigned a random number. Children with the highest number were invited to participate sequentially until the desired sample size was achieved in each cluster.

### Eligibility, recruitment and consent

Children were eligible for inclusion if aged 4 to 6 years and resident in a selected village at the time of the study. The age inclusion criteria were chosen to estimate the prevalence of disease at school-entry age. Children were excluded if they had died or moved since the initial census, or if they did not provide assent to participation. Written informed consent was obtained from the guardian of all participants by a field worker fluent in the local language, Chichewa.

### Sample size

We calculated that approximately 270 participants would be sufficient to estimate the prevalence of CSOM with a precision of +/-3% at a 5% alpha level. A design effect of 1.2 and a prevalence of 6% were assumed after a review of prevalence surveys in comparable settings and ages.[4,11–20]

### Data collection

A structured interview was performed using a standardised questionnaire (S1 Appendix). Demographic information and potential risk factors for CSOM were collected from the guardian. Previous measles or meningitis, vaccination history and HIV status were confirmed in the participant’s health passport. A history of ear pain, discharge and fever were elicited from the patient and guardian.

A hearing assessment was performed using a HearScreen^TM^ screening audiometer (Hearscreen, Pretoria, South Africa), with supra-aural Sennheiser HD280 headphones (Sennheiser, Wedemark, Germany), calibrated to ANSI S3.6 standard.[8,28] Screening audiometry was undertaken according to the HearScreen validation study protocol.[8,29] Each participant was tested using a 25 decibel tone at 1000Hz, 2000Hz and 4000Hz. Hearing impairment was classified as failure to hear a pure tone of 25 dB or greater at 1, 2 or 4kHz. We omitted 500Hz due to potential for ambient noise interference during fieldwork.

Tests were conducted in the quietest available room in each community. Ambient noise levels were monitored and tests repeated if maximum permissible noise levels were exceeded (49, 51, and 57 dB for 1, 2 and 4kHz, respectively).[28] Participants were categorised as having hearing impairment if unable to hear one or more frequency in either ear. Tests were repeated in participants who failed the first test. Screening audiometry was performed prior to cerumen management.

**Fig 1:**
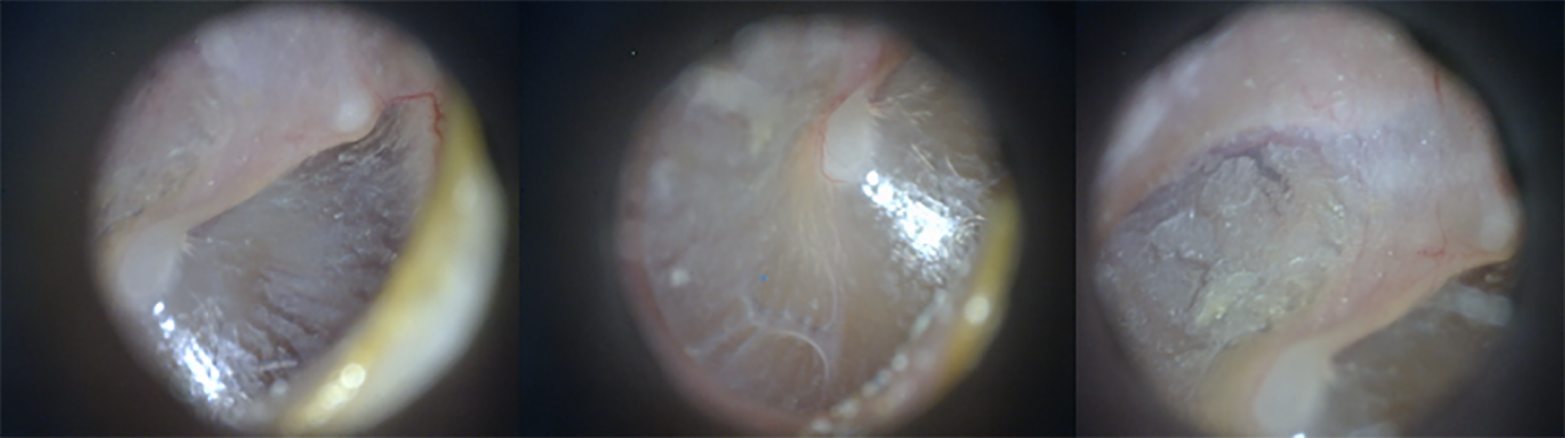
Serial images of the right tympanic membrane of a healthy volunteer captured by the clinical officer using the Dino-Lite^TM^ MEDL4E video otoscope.

Tympanic membrane examination was performed by a Clinical Health Officer using a Dino-Lite^TM^ MEDL4E otoscope (PC Werth, London, UK) following study-specific training. Fig 1 illustrates the tympanic membrane image quality achieved with the Dino-lite^TM^ otoscope. Where appropriate, cerumen management was performed under direct vision using a Bionix^TM^ lighted ear curette (Bionix, California, USA). Cerumen removal was performed to tolerance and halted in the event of discomfort or withdrawal of assent.

### Clinical management and confirmation of CSOM cases

All participants with ear pathology received appropriate medical treatment at diagnosis. All children with CSOM were given assistance to attend the tertiary otolaryngology centre at Queen Elizabeth Central Hospital, Blantyre, Malawi for specialist assessment. Guardians were compensated for associated financial and opportunity costs.

### Diagnostic assessment

Anonymised data were viewed remotely by two otolaryngologists (WM, SD) with 20 years’ cumulative experience. Assessors commented on image quality and assigned a diagnosis to each ear using a structured image interpretation tool. Assessors had access to tympanic membrane images, screening audiometry results, and clinical findings. Diagnoses were assigned as per the WHO classification of ear and hearing disorders and are summarised in Table 1. Assessments were done independently in duplicate, with diagnostic disagreements resolved through discussion.[30]

**Table 1:** WHO classification of middle ear disorders. **Source: Smith & Mackenzie 1999**[30].

Diagnosis	Features
Normal	Normal appearances of the tympanic membrane
Foreign Body	Foreign body
Cerumen Impaction	Wax plug obstructing the tympanic membrane unsuitable for removal in the field
Acute Otitis Media	Ear pain or discharge for lasting less than two weeks with a red or opaque tympanic membrane with moderate-severe bulging
Serous Otitis Media	Retracted and dull tympanic membrane
Chronic Suppurative Otitis Media	Persistent purulent discharge lasting for longer than 2 weeks in conjunction with ottorhoea in the external auditory meatus and a perforated tympanic membrane on examination.
Dry Perforation	Perforated tympanic membrane without evidence
Healed Perforation	Healed perforation of the tympanic membrane

Each participant was assigned a single middle ear diagnosis. A normal diagnosis was assigned if participants had healthy tympanic membranes bilaterally. Where participants had a differing diagnosis in each ear, the most clinically serious diagnosis was taken: CSOM, followed by dry perforation, serous otitis media, acute otitis media, foreign body and cerumen impaction.

### Statistical analysis

Prevalence estimates were calculated as a weighted average of the cluster level means and reported alongside 95% confidence intervals. Weighting was determined by the sampling fraction with additional post-hoc weighting at an individual level to account for sex. Due to the narrow age inclusion criteria, analyses were not weighted for age. Confidence intervals were constructed assuming a normal distribution with standard errors calculated from a bootstrapping without replacement approach, modified for two stage sampling.[31] 100,000 bootstrap samples were taken. The design effect is reported as the ratio of the calculated variance to that computed under simple random sampling.

Associations of CSOM were evaluated using univariate analyses with no formal statistical testing. Relative risk estimates for categorical variables were calculated as the ratio of the sample means between each group. A binomial generalised linear model with a log link function was fitted to continuous variables without post-hoc weighting. The relative risk is reported as the exponential of the estimated coefficient. Confidence intervals for relative risk estimates were calculated assuming a log-normal distribution with standard errors estimated from the bootstrap samples above. A constant of a half was added to each cell entry when calculating relative risk for each bootstrap replicate to avoid 0 and infinity estimates.[32] Patients with missing data were assumed to be missing at random and listwise deletion was applied. Data were analysed using “R” version 3.2.0.

### Ethical approval, governance and data sharing

Ethical approval was granted by the science and ethics review committees of the Liverpool School of Tropical Medicine and the Malawi College of Medicine. The protocol was prospectively registered with ClinTrials.gov (Protocol ID NCT02779907). The full anonymized dataset are available in S2 Appendix.

## Results

**Fig 2:**
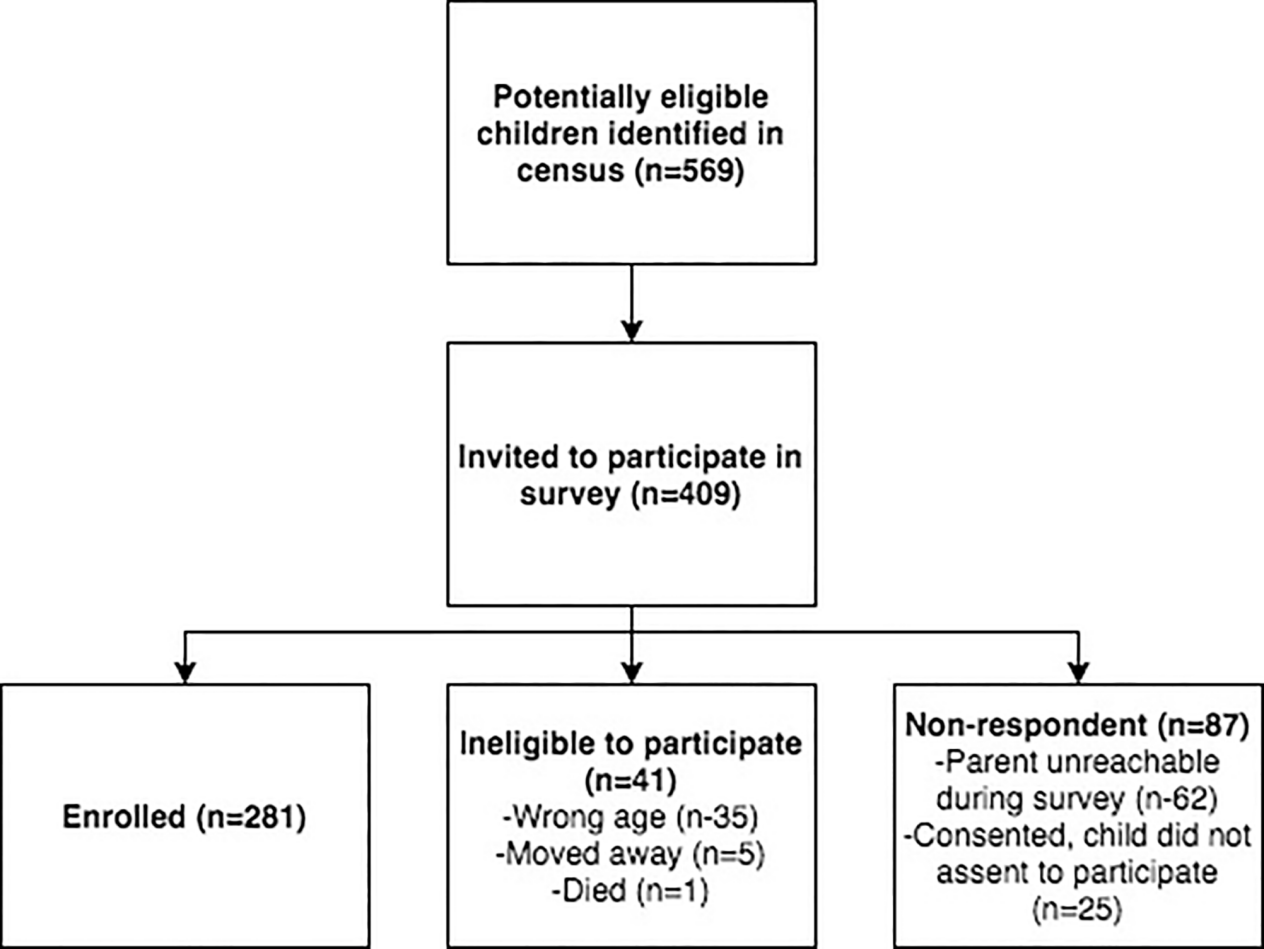
Flowchart of participant enrolment.

We recruited 281 children aged 4 to 6 years from 10 villages between 11^th^ May and 23^rd^ June 2016 (Fig 2). Of 569 children originally identified, 41 were found to be ineligible at the time of fieldwork. Five had moved, one had died and thirty-five did not meet the age inclusion criteria. We examined 281 of 528 children potentially eligible to participate giving a sampling fraction of 53.2%. The response rate was 76.4%, defined as the proportion of eligible individuals invited to participate who completed the survey. S1 Table details the sampling fraction and response rate by cluster. Of non-respondents, 62 children could not be found or were unable to attend during the fieldwork. Twenty-five children fulfilled the eligibility criteria, but did not assent to examination.

### Baseline demographics

The median age of children included in the study was 5.2 years (IQR 4.6–5.8) with 42.0, 43.4 and 14.6% aged 4, 5 and 6 respectively. There were slightly more male (54.1%) than female participants. The age and gender distribution of our sample was comparable to 2008 census data[25] with the exception of 6 year olds who were under-represented in our sample. S2 Table provides detailed comparison of study demographics compared to 2008 census data.

### Tympanic membrane image quality

In total, 562 ears were assessed in 281 participants. Image quality was rated as excellent in 15.3% (86/562), good in 72.4% (407/562) and inadequate in 3.2% (18/562). Cerumen impaction prevented visualisation of the tympanic membrane in 9.1% (51/562).

### Prevalence of middle ear disease

CSOM was diagnosed in 15/281 (5.3%) of participants, with 4/15 (26.7%) having bilateral disease. In all cases the diagnosis was confirmed by a third otolaryngologist on referral to the otolaryngology centre. No participant displayed clinical features of mastoiditis or cholesteatoma. The weighted prevalence of CSOM was estimated at 5.4% (95% CI, 2.2–8.6, design effect (DEFF) 1.49).

Table 2 shows the tympanic membrane diagnosis for each participant. Cerumen impaction was the most commonly seen abnormality, occurring in 40/281 (14.2%) of the sample. Sixty eight percent of participants (192/281) had bilateral normal tympanic membranes.

**Table 2:** Final tympanic membrane diagnosis for all study participants.

Middle Ear Diagnosis	n (%)
Normal Ears bilaterally	192 (68.3)
Cerumen Impaction	40 (14.2)
Acute otitis media	8 (2.8)
Serous otitis media	10 (3.6)
Chronic suppurative otitis media	15 (5.3)
Dry Perforation	1 (0.4)
Foreign Body	1 (0.4)
Unable to assign diagnosis (poor image quality)	14 (5.0)
Missing	0 (0)
Total	281 (100)

### Hearing impairment

Audiometry data were complete for all but 2/281 patients. Background ambient noise was monitored throughout the study and found to be an average of 45.3 dB. Median screening audiometry time was 123 seconds (IQR 73–171). Hearing impairment at 25 dB or greater hearing level in at least one ear was observed in 64/279 participants, with a weighted prevalence estimate of 24.5% (95% CI 15.0–33.9, DEFF 3.5). Bilateral hearing impairment was observed in 32/279 participants, with a weighted prevalence of 12.5% (95% CI 6.2–16.9, DEFF 1.8). Of those with hearing impairment, 46.9% (30/64) had an identifiable and potentially reversible aetiology (Table 3). The commonest diagnosis was CSOM which accounted for 15.6% (10/64) of cases of hearing impairment.

**Table 3:** Tympanic membrane diagnoses in children with hearing impairment >25 dB.

Tympanic membrane diagnosis	n (%)
Acute otitis media	4 (6.3)
Serous otitis media	6 (9.3)
Chronic suppurative otitis media	10 (15.6)
Dry perforation	1 (1.6)
Cerumen impaction	7 (10.9)
Normal	36 (56.3)
Poor TM image quality	8 (12.5)
Missing	0 (0)
Total	64 (100)

### Associations of CSOM

Univariate analysis for potential associations of CSOM was limited by the low number of CSOM cases observed. There was a trend in increasing risk of CSOM with increasing age and sleeping in a house with >2 other children (Table 4). There was no trend towards difference in CSOM prevalence between the intervention and control groups of CAPS.

**Table 4:** Univariate analysis exploring potential associations of Chronic suppurative otitis media.

Risk factor		No CSOM (n = 266)	CSOM (n = 15)	CSOM Prevalence estimate	Relative Risk (95% CI)
**Median (IQR)**					
**Age (years)**	Ascending	5.08 (4.58–5.73)	5.58 (4.92–5.96)	-	2.08 (0.67–6.52)
**Mid-upper arm circumference**		15.50 (15.00–16.40)	15.50 (14.55–16.35)	-	0.93 (0.61–1.41)
**N (column %)**					
**Sex**	Male	143 (53.8%)	9 (60.0%)	5.44	1
	Female	123 (46.2%)	6 (40.0%)	5.09	0.93 (0.21–4.13)
**Number of other children <12 years in household**	2 or fewer	246 (92.5)	13 (86.7)		1
	3 or more	20 (7.5)	2 (13.3)		1.85 (0.28–12.18)
**Primary household cooking appliance**	Open fire	125 (47.0%)	6 (40.0%)	4.61	1
	Efficient cookstove	141 (53.0%)	9 (60.0%)	5.87	1.27 (0.59–2.72)
**Previous Measles**	No	260 (97.7%)	14 (93.3%)	5.08	1
	Yes	6 (2.3%)	1 (6.7%)	13.12	2.58 (0.42–26.54)
**HIV Status**	Not infected	262 (98.9%)	15 (100.0%)	5.28	-
	Infected	3 (1.1%)	0	0	-
**Completed pneumococcal vaccination course**	No	11 (4.1%)	0	0	-
	Yes	255 (95.9%)	15 (100.0%)	5.28	-

## Discussion

The prevalence of CSOM in our study population is estimated at 5.4% (95% CI, 2.2–8.6), placing this rural Malawian population in the highest WHO category for disease burden.[2] The reasons for this are likely multifactorial. Surveys of CSOM prevalence in sub-Saharan Africa have consistently reported higher estimates in rural populations when compared to urban populations.[13,18,20,33] Associations of otitis media in this setting include low socioeconomic status, crowding, malnutrition and exposure to wood smoke.[12,23,24] Exposure to these factors may be greater amongst the rural poor. In our study area, extreme poverty, food insecurity and use of traditional indoor cooking methods reliant on biomass fuel result in substantial exposure to these risk factors, and may contribute to a high disease burden.

We report a prevalence of unilateral hearing impairment at 25dB of 24.5% (95% CI 15.0–33.9) and bilateral hearing impairment of 12.5% (95% CI 6.2–16.9). In this study we provide hearing impairment estimates from screening audiometry. In the validation study, HearScreen was compared to diagnostic audiometry using a pure tone average of >25 dB at 0.5, 1,2 and 4 kHz. HearScreen and was shown to have a sensitivity of 75% and a specificity of 98.5%.[28] We utilized the HearScreen validation study protocol and tested participants of similar ages in a comparable setting. Thus, our prevalence estimate may be interpreted as a minimum indicative level of hearing impairment in the study population.

Our hearing impairment estimate is relatively high when compared to a recent systematic review of hearing impairment in Africa.[3] This review identified two community-based studies of all ages which defined hearing impairment at a pure tone average at 25 dB. These studies reported the prevalence of hearing impairment of 16% and 17.9%, respectively.[3] A further six school-based studies defining hearing impairment as a as pure tone average at 25 dB were identified, reporting a median prevalence of unilateral hearing impairment of 7.7% (Range 2.4–21.3), and no age effect identified.[3] It is possible that higher prevalence estimates in our and other community-based surveys may reflect a systematic bias of school-based surveys due to exclusion of school non-attenders. However, we encountered high levels of ambient noise during field work, and were unable to perform Otoacousic emission testing for younger participants. Thus, hearing impairment may be over-estimated in our study. Additionally, we performed cerumen management after screening audiometry, and therefore our prevalence estimate is likely higher than studies which undertook this procedure prior to audiometry.

Of participants with hearing impairment, half had evidence of middle ear disease. Identification and management of CSOM and bilateral hearing impairment at 25 dB is particularly important in this age group, as both have been associated with decreased academic attainment in primary school children across a range of settings.[4,34–36] The educational environment of Malawi is challenging, with just 49.8% of children completing primary schooling.[26] Although the reasons for this are complex, the burden of middle ear disease and subsequent hearing impairment amongst 4–6 year-olds identified in this survey may represent a barrier to academic attainment.

Throughout sub-Saharan Africa, primary healthcare outside large cities is often delivered by practitioners with limited training and equipment to diagnose and manage ear and hearing disorders. In this context, provision of otolaryngology services to rural communities presents a substantial challenge. In this study, we utilised a telehealth approach, with a protocolised ear assessment performed by a non-specialist Clinical Officer and evaluated remotely by an Otolaryngologist. This approach links centralised specialist otolaryngology services with rural communities, where the burden of middle ear disease is highest. A telehealth approach, piloted for both otoscopy and screening audiometry in rural South Africa, has proven promising in terms of clinical validity and acceptability.[10,28,37] However, evidence as to the operational and cost effectiveness of such an intervention is currently lacking, and further evaluation is needed.

The Dino-Lite^TM^ MEDL4E otoscopes used in this study represent an affordable option which may be suited to use in low-resource settings. We found the otoscopes to be reliable and produced diagnostic quality images. However, due to high magnification levels, multiple photographs were required to image the whole tympanic membrane when utilising paediatric specula. This may be problematic where assent is withdrawn and may have contributed to poor image quality recorded in a minority of cases.

This study has limitations. We did not perform tympanometry to confirm diagnoses of acute and serous otitis media, and pneumatic otoscopy was considered unfeasible due to the asynchronous telehealth approach. These factors are likely to lead to a decreased diagnostic accuracy for acute and serous otitis media and the true prevalence may be higher than measured.[38] Additionally, cerumen impaction was diagnosed in 14.2% of cases, and poor image quality in 5%. Thus, the true prevalence of other middle ear diagnoses may be higher than measured in this survey.

The confidence intervals around our prevalence estimate for CSOM are slightly broader than those specified in the sample size. We had planned to use probability proportional to size sampling to select the village clusters, however recent flooding and food insecurity in the area resulted in substantial population movements, invalidating previously available census data on village sizes. To ensure a probability sample, we selected the village clusters by simple random sampling, which resulted in unequal cluster sizes and likely increased the sampling design effect and uncertainty around our prevalence estimate. Additionally, we sampled more 4 and 5 year-olds than 6 year-olds. Children commence schooling aged 6 and it may be these children were more likely to be a source of non-response. Due to the narrow age-inclusion criteria, this is not thought to substantially affect the prevalence estimate.

The study was not powered to formally assess potential associations of CSOM, and analysis was limited by the low number of CSOM cases observed. Crowding and increasing age appeared to display a positive effect size. There was no difference observed in CSOM prevalence in CAPS intervention versus control households. Possible explanations for this include inadequate observed case numbers, inadequate emissions reductions from the cookstoves in the field,[22] inadequate adoption and declining use of the intervention over time, and exposure to other sources of air pollution other than from cooking.

In conclusion, we found a high burden of CSOM and other potentially treatable ear diseases and preventable hearing impairment in our sample of school-entry age children in rural Malawi. Since CSOM can adversely affect academic performance and development there are substantial public health implications of these findings which, we suggest, are generalizable to surrounding areas with similar geography and development indicators. The identification and management of middle ear disease and hearing impairment represent major unmet needs in this vulnerable population.

## Supporting information

S1 Appendix Data collection tool. (DOCX)

S2 Appendix Full anonymised dataset. (XLSX)

S1 Table Sampling fraction and response rate by cluster. (DOCX)

S2 Table Comparison of study demographics to 2008 census data. (DOCX)

## References

[1] Monasta L, Ronfani L, Marchetti F, Montico M, Vecchi Brumatti L, Bavcar A, et al Burden of disease caused by otitis media: systematic review and global estimates. PLoS One. Public Library of Science; 2012;7: e36226 doi: 10.1371/journal.pone.0036226 22558393 10.1371/journal.pone.0036226 PMC3340347

[2] WHO. Chronic suppurative otitis media—Burden of Illness and Management Options [Internet]. WHO Library Cataloguing-in-Publication Data. 2004. doi: 10.1016/j.amjoto.2007.09.002

[3] Mulwafu W, Kuper H, Ensink RJH. Prevalence and causes of hearing impairment in Africa. Trop Med Int Health. 2015;21: 158–165. doi: 10.1111/tmi.12640 26584722 10.1111/tmi.12640

[4] Olatoke F, Ologe FE, Nwawolo CC, Saka MJ. The prevalence of hearing loss among schoolchildren with chronic suppurative otitis media in Nigeria, and its effect on academic performance. Ear Nose Throat J. 2008;87: E19 19105130

[5] Dubey SP, Larawin V. Complications of Chronic Suppurative Otitis Media and Their Management. Laryngoscope. John Wiley & Sons, Inc.; 2007;117: 264–267. doi: 10.1097/01.mlg.0000249728.48588.22 17277619 10.1097/01.mlg.0000249728.48588.22

[6] Thornton D, Martin TPC, Amin P, Haque S, Wilson S, Smith MCF. Chronic suppurative otitis media in Nepal: ethnicity does not determine whether disease is associated with cholesteatoma or not. J Laryngol Otol. 2011;125: 22–6. doi: 10.1017/S0022215110001878 20875194 10.1017/S0022215110001878

[7] Mulwafu W, Nyirenda TE, Fagan JJ, Bem C, Mlumbe K, Chitule J. Initiating and developing clinical services, training and research in a low resource setting: the Malawi ENT experience. Trop Doct. 2014;44: 135–139. doi: 10.1177/0049475514524393 24569097 10.1177/0049475514524393

[8] Mahomed-Asmail F, Swanepoel DW, Eikelboom RH, Myburgh HC, Hall J. Clinical Validity of hearScreen^TM^ Smartphone Hearing Screening for School Children. Ear Hear. 37: e11–7. doi: 10.1097/AUD.0000000000000223 26372265 10.1097/AUD.0000000000000223

[9] Biagio L, Swanepoel DW, Adeyemo A, Iii JWH, Vinck B. Asynchronous Video-Otoscopy with a Telehealth Facilitator. Telemed J E Health. Mary Ann Liebert, Inc. 140 Huguenot Street, 3rd Floor New Rochelle, NY 10801 USA; 2012; 1–3. doi: 10.1089/tmj.2012.9999 23384332 10.1089/tmj.2012.0161

[10] Lundberg T, Biagio L, Laurent C, Sandström H, Swanepoel DW. Remote evaluation of video-otoscopy recordings in an unselected pediatric population with an otitis media scale. Int J Pediatr Otorhinolaryngol. 2014;78: 1489–1495. doi: 10.1016/j.ijporl.2014.06.018 25017799 10.1016/j.ijporl.2014.06.018

[11] Westerberg BD, Skowronski DM, Stewart IF, Stewart L, Bernauer M, Mudarikwa L. Prevalence of hearing loss in primary school children in Zimbabwe. Int J Pediatr Otorhinolaryngol. 2005;69: 517–525. doi: 10.1016/j.ijporl.2004.11.020 15763291 10.1016/j.ijporl.2004.11.020

[12] Amusa YB, Ijadunola IK, Onayade OO. Epidemiology of otitis media in a local tropical African population. West African journal of medicine. 2005 pp. 227–230. 16276700 10.4314/wajm.v24i3.28202

[13] Simoes A. E, Kiio F., Carosone-Link J. P, et al Otitis Media and Its Sequelae in Kenyan Schoolchildren. J Pediatr Infect Dis Soc. 2015; 1–10. doi: 10.1093/jpids/piv038 26407271 10.1093/jpids/piv038 PMC5181359

[14] Aduda DSO, Macharia IM, Mugwe P, Oburra H, Farragher B, Brabin B, et al Bacteriology of chronic suppurative otitis media (CSOM) in children in Garissa district, Kenya: A point prevalence study. Int J Pediatr Otorhinolaryngol. Elsevier Ireland Ltd; 2013;77: 1107–1111. doi: 10.1016/j.ijporl.2013.04.011 23711391 10.1016/j.ijporl.2013.04.011

[15] Hatcher J, Smith A, Mackenzie I, Thompson S, Bal I, Macharia I, et al A prevalence study of ear problems in school children in Kiambu district, Kenya, May 1992. Int J Pediatr Otorhinolaryngol. 1995;33: 197–205. doi: 10.1016/0165-5876(95)01209-5 8557476 10.1016/0165-5876(95)01209-5

[16] Swart SM, Lemmer R, Parbhoo JN, Prescott CAJ. A survey of ear and hearing disorders amongst a representative sample of Grade 1 schoolchildren in Swaziland. Int J Pediatr Otorhinolaryngol. 1995;32: 23–34. doi: 10.1016/0165-5876(94)01109-B 7607818 10.1016/0165-5876(94)01109-b

[17] Prescott CAJ, Kibel MA. Ear and hearing disorders in rural grade 2 (Sub B) schoolchildren in the western Cape. South African Med J. 1991;79: 90–93. 1989096

[18] Adebola SO, Ayodele SO, Oyelakin OA, Babarinde JA, Adebola OE. Pre-school hearing screening: Profile of children from Ogbomoso, Nigeria. Int J Pediatr Otorhinolaryngol. Elsevier Ireland Ltd; 2013;77: 1987–1991. doi: 10.1016/j.ijporl.2013.09.019 24139592 10.1016/j.ijporl.2013.09.019

[19] Bastos I, Mallya J, Ingvarsson L, Reimer?? ke, Andr??asson L. Middle ear disease and hearing impairment in northern Tanzania. A prevalence study of schoolchildren in the Moshi and Monduli districts. Int J Pediatr Otorhinolaryngol. 1995;32: 1–12. doi: 10.1016/0165-5876(94)01904-C 7607816 10.1016/0165-5876(94)01904-c

[20] Minja BM, Machemba A. Prevalence of otitis media, hearing impairment and cerumen impaction among school children in rural and urban Dar es Salaam, Tanzania. Int J Pediatr Otorhinolaryngol. 1996;37: 29–34. doi: 10.1016/0165-5876(96)01363-8 8884404 10.1016/0165-5876(96)01363-8

[21] Mortimer K, Ndamala CB, Naunje AW, Malava J, Katundu C, Weston W, et al A cleaner burning biomass-fuelled cookstove intervention to prevent pneumonia in children under 5 years old in rural Malawi (the Cooking and Pneumonia Study): a cluster randomised controlled trial. Lancet. 2017;389: 167–175. doi: 10.1016/S0140-6736(16)32507-7 27939058 10.1016/S0140-6736(16)32507-7 PMC5783287

[22] Wathore R, Mortimer K, Grieshop AP. In-Use Emissions and Estimated Impacts of Traditional, Natural- and Forced-Draft Cookstoves in Rural Malawi. Environ Sci Technol. 2017; acs.est.6b05557. doi: 10.1021/acs.est.6b05557 28060518 10.1021/acs.est.6b05557 PMC5323018

[23] da Costa JL, Navarro A, Neves JB, Martin M. Household wood and charcoal smoke increases risk of otitis media in childhood in Maputo. Int J Epidemiol. 2004;33: 573–578. doi: 10.1093/ije/dyh071 15105407 10.1093/ije/dyh071

[24] Lasisi AO, Olaniyan FA, Muibi SA, Azeez IA, Abdulwasiu KG, Lasisi TJ, et al Clinical and demographic risk factors associated with chronic suppurative otitis media. Int J Pediatr Otorhinolaryngol. 2007;71: 1549–54. doi: 10.1016/j.ijporl.2007.06.005 17643499 10.1016/j.ijporl.2007.06.005

[25] National Statistical Office. Malawi Population and Housing Census [Internet]. 2008. Available: http://www.nsomalawi.mw/2008-population-and-housing-census.html

[26] National Statistics Office. Malawi Demographic and Health Survey. 2010; Available: http://www.dhsprogram.com/pubs/pdf/FR247/FR247.pdf

[27] Famine early warning systems network. Malawi Food Security Outlook June 2016—January 2017 [Internet]. 2016. Available: http://www.fews.net/sites/default/files/documents/reports/Final_MW_FSO_2016_06.pdf

[28] Swanepoel DW, Myburgh HC, Howe DM, Mahomed F, Eikelboom RH. Smartphone hearing screening with integrated quality control and data management. Int J Audiol. Taylor & Francis; 2014; 10.3109/14992027.2014.920965 24998412

[29] Swanepoel DW. Clinical Validity of hearScreen Smartphone Hearing Screening for School Children. Ear Hear. 2016;37: e11–e17. doi: 10.1097/AUD.0000000000000223 26372265 10.1097/AUD.0000000000000223

[30] Smith A, Mackenzie I. WHO Ear and Hearing Disorders Survey Protocol [Internet]. 1999. Available: http://www.who.int/pbd/deafness/activities/epidemiology_economic_analysis/en/

[31] Sitter RR. Comparing three bootstrap methods for survey data. Can J Stat / La Rev Can Stat. 1992;20: 135–154. Available: http://dx.doi.org/10.2307/3315464%5Cnpapers2://publication/doi/10.2307/3315464

[32] Haldane J. The estimation and significance of the logarithm of a ratio of frequencies. Ann Hum Genet. 1956;20: 309–311. 13314400 10.1111/j.1469-1809.1955.tb01285.x

[33] Ologe FE, Nwawolo CC. Chronic suppurative ototis media in school pupils in Nigeria. East Afr Med J. 2004;80: 12–16. doi: 10.4314/eamj.v80i3.8681 10.4314/eamj.v80i3.8681 12762427

[34] Khairi Md Daud M, Noor RM, Rahman NA, Sidek DS, Mohamad A. The effect of mild hearing loss on academic performance in primary school children. Int J Pediatr Otorhinolaryngol. 2010;74: 67–70. doi: 10.1016/j.ijporl.2009.10.013 19913305 10.1016/j.ijporl.2009.10.013

[35] Bess FH, Dodd-Murphy J, Parker RA. Children with minimal sensorineural hearing loss: prevalence, educational performance, and functional status. Ear Hear. 1998;19: 339–54. 9796643 10.1097/00003446-199810000-00001

[36] Blair J, Peterson ME, Vichweg SH. The effects of mild sensorineural hearing loss on academic performance of young school-age children. Volta Rev. 1985;93: 87–93.

[37] Biagio L, Swanepoel DW, Adeyemo A, Hall JW, Vinck B. Asynchronous video-otoscopy with a telehealth facilitator. Telemed J E Health. 2013;19: 252–8. doi: 10.1089/tmj.2012.0161 23384332 10.1089/tmj.2012.0161

[38] Shekelle P, Takata G, Chan L, Mangione-Smith R, Corley P, Morphew T. AHRQ Evidence Report: Diagnosis, Natural History, and Late Effects of Otitis Media With Effusion [Internet]. Rockville, MD: Agency for Healthcare Research and Quality; 2003 Available: http://www.ncbi.nlm.nih.gov/books/NBK11875/10.1037/e439822005-001 PMC4781261 12945555

