# Supplementary figures and images for: Prevalence of paediatric chronic suppurative otitis media and hearing impairment in rural Malawi: A cross-sectional survey
Source: PLoS One. 2017 Dec 21;12(12):e0188950. doi: 10.1371/journal.pone.0188950 (PMC5739401; doi:10.1371/journal.pone.0188950)

**S1 Supplementary Appendix.** Data collection tool

***
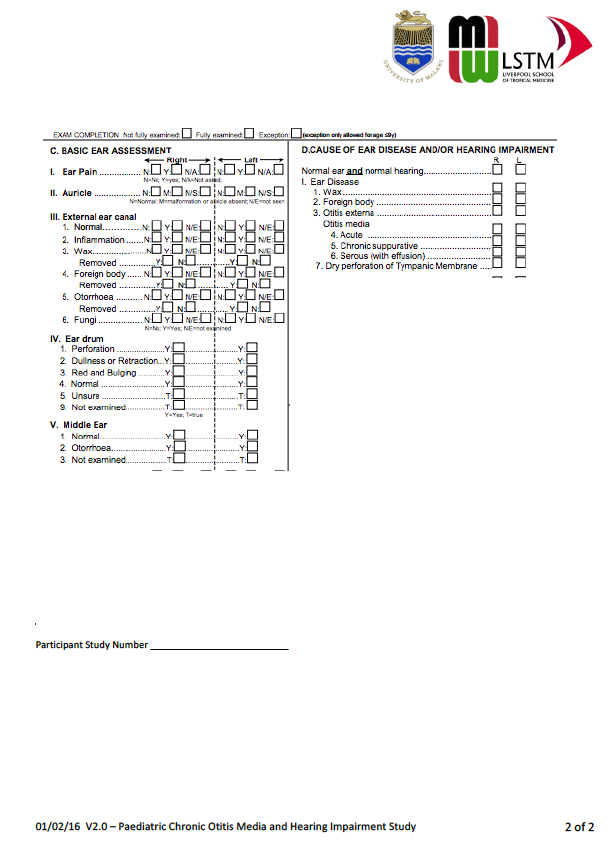
***

Supplement: S1 Appendix — (DOCX) [file pone.0188950.s001.docx]
