# Supplementary material for: Prevalence of paediatric chronic suppurative otitis media and hearing impairment in rural Malawi: A cross-sectional survey
Source: PLoS One. 2017 Dec 21;12(12):e0188950. doi: 10.1371/journal.pone.0188950 (PMC5739401; doi:10.1371/journal.pone.0188950)
Supplement: S1 Table — (DOCX) [file pone.0188950.s003.docx]

**S3 Supplementary Appendix. Survey sampling results by village cluster**

|  |  |  |  |  |  |  |  |  | | | |  | |  | | | |  |  |
| --- | --- | --- | --- | --- | --- | --- | --- | --- | --- | --- | --- | --- | --- | --- | --- | --- | --- | --- | --- |
| **Village Cluster** | **Total identified at initial Survey** | **Total Eligible at time of survey** | **Number Requested** | **Number eligible** | **Ineligible** | | | | | **Non-Respondent** | | | | | **Number Enrolled** | **Sampling Fraction** | **Participation Rate** | | |
|  |  |  |  |  | **Wrong Age** | **Died** | **Moved Away** | | **TOTAL** | **Unreachable** | **Child did not assent** | | **TOTAL** | |  |  |  |  |  |
| 1 | 23 | 23 | 16 | 16 | 0 | 0 | 0 | | 0 | 5 | 0 | | 5 | | 11 | 47.8 | 68.8 | | |
| 2 | 19 | 18 | 17 | 16 | 0 | 0 | 1 | | 1 | 3 | 4 | | 7 | | 9 | 50.0 | 56.3 | | |
| 3 | 52 | 51 | 34 | 33 | 1 | 0 | 0 | | 1 | 6 | 1 | | 7 | | 26 | 51.0 | 78.8 | | |
| 4 | 22 | 20 | 17 | 15 | 2 | 0 | 0 | | 2 | 3 | 0 | | 3 | | 12 | 60.0 | 80.0 | | |
| 5 | 78 | 73 | 52 | 47 | 5 | 0 | 0 | | 5 | 4 | 2 | | 6 | | 41 | 56.2 | 87.2 | | |
| 6 | 74 | 67 | 50 | 43 | 5 | 0 | 2 | | 7 | 6 | 6 | | 12 | | 31 | 46.3 | 72.1 | | |
| 7 | 92 | 83 | 67 | 58 | 8 | 0 | 1 | | 9 | 10 | 2 | | 12 | | 46 | 55.4 | 79.3 | | |
| 8 | 111 | 100 | 89 | 78 | 10 | 1 | 0 | | 11 | 15 | 4 | | 19 | | 59 | 59.0 | 75.6 | | |
| 9 | 82 | 81 | 53 | 52 | 1 | 0 | 0 | | 1 | 8 | 5 | | 13 | | 39 | 48.1 | 75.0 | | |
| 10 | 16 | 12 | 14 | 10 | 3 | 0 | 1 | | 4 | 2 | 1 | | 3 | | 7 | 58.3 | 70.0 | | |
| Totals | 569 | 528 | 409 | 368 | 35 | 1 | 5 | | 41 | 62 | 25 | | 87 | | 281 | 53.2 | 76.4 | | |
