# Supplementary material for: Prevalence of paediatric chronic suppurative otitis media and hearing impairment in rural Malawi: A cross-sectional survey
Source: PLoS One. 2017 Dec 21;12(12):e0188950. doi: 10.1371/journal.pone.0188950 (PMC5739401; doi:10.1371/journal.pone.0188950)
Supplement: S2 Table — (DOCX) [file pone.0188950.s004.docx]

**S4 Supplementary Appendix. Survey population composition compared to regional and national census data.**^22^

|  | Current Study | 2008 Census – National figures |
| --- | --- | --- |
| Age (years) | n (%) | n (%) |
| 4 | 118 (42.0) | 478003 (36.8) |
| 5 | 122 (43.4) | 435984 (33.6) |
| 6 | 41 (14.6) | 383178 (29.5) |
| Missing | 0 (0) | 0 (0) |
| Total | 281 (100) | 281 (100) |
|  | **Current Study** | **2008 Census - Chikhwawa figures** |
| Sex | n (%) | n (%) |
| Female | 129 (45.9) | 218628 (50.3) |
| Male | 152 (54.1) | 216020 (49.7) |
| Missing | 0 (0) | 0 (0) |
| Total | 281 (100) | 281 (100) |
